# Astaxanthin attenuates total body irradiation-induced hematopoietic system injury in mice via inhibition of oxidative stress and apoptosis

**DOI:** 10.1186/s13287-016-0464-3

**Published:** 2017-01-23

**Authors:** Xiao-Lei Xue, Xiao-Dan Han, Yuan Li, Xiao-Fei Chu, Wei-Min Miao, Jun-Ling Zhang, Sai-Jun Fan

**Affiliations:** 1 Tianjin Key Lab of Radiation Medicine and Molecular Nuclear Medicine, Institute of Radiation Medicine, Peking Union Medical College and Chinese Academy of Medical Sciences, Tianjin, 300192 China; 2 State Key Laboratory of Experimental Hematology, Institute of Hematology and Blood Disease Hospital, Peking Union Medical College and Chinese Academy of Medical Sciences, Tianjin, 300020 China

**Keywords:** Astaxanthin, Ionizing radiation, Hematopoietic stem cells, Reactive oxygen species, Cell apoptosis

## Abstract

**Background:**

The hematopoietic system is especially sensitive to total body irradiation (TBI), and myelosuppression is one of the major effects of TBI. Astaxanthin (ATX) is a powerful natural anti-oxidant with low toxicity. In this study, the effect of ATX on hematopoietic system injury after TBI was investigated.

**Methods:**

Flow cytometry was used to detect the proportion of hematopoietic progenitor cells (HPCs) and hematopoietic stem cells (HSCs), the level of intracellular reactive oxygen species (ROS), expression of cytochrome C, cell apoptosis, and NRF2-related proteins. Immunofluorescence staining was used to detect Nrf2 translocation. Western blot analysis was used to evaluate the expression of apoptotic-related proteins. Enzymatic activities assay kits were used to analyze SOD2, CAT, and GPX1 activities.

**Results:**

Compared with the TBI group, ATX can improve radiation-induced skewed differentiation of peripheral blood cells and accelerate hematopoietic self-renewal and regeneration. The radio-protective effect of ATX is probably attributable to the scavenging of ROS and the reduction of cell apoptosis. These changes were associated with increased activation of Nrf2 and downstream anti-oxidative proteins, and regulation of apoptotic-related proteins.

**Conclusions:**

This study suggests that ATX could be used as a potent therapeutic agent to protect the hematopoietic system against TBI-induced bone marrow suppression.

## Background

Accidental exposure to moderate or high doses of radiation poses a significant threat to human health, especially the hematopoietic system [1]. The hematopoietic system is the most vulnerable system to the damaging effects of total body irradiation (TBI), and doses greater than 1 Gy induce bone marrow (BM) cell injury [2]. BM suppression is also the main symptom of hematopoietic systemic injury seen in the clinic when patients receive traditional cancer therapy, such as radiotherapy, and this can cause high mortality and morbidity which worsens the outcome of cancer treatment [3, 4]. TBI-induced myelosuppression, which presents mainly as pancytopenia, is due to apoptosis of the rapidly proliferating hematopoietic progenitor cells (HPCs) and the relatively quiescent hematopoietic stem cells (HSCs) [5]. Thus, efforts to reduce apoptosis of HSCs not only help to attenuate TBI-induced HSC depletion, but also relieve TBI-induced BM injury in the hematopoietic system.

Under steady-state conditions HSCs are quiescent and serve as a reserve to protect the hematopoietic system from exhaustion against various stress conditions [6]. However, exposure to ionizing radiation (IR) can generate large amounts of reactive oxygen species (ROS) in the cytosol of mammalian cells, which results in severe oxidative stress. Although ROS are required for the physiological function of cells, overproduction of these molecules damages cellular components, such as proteins, lipids, and DNA, particularly through DNA double-strand breaks (DSBs), which finally results in hematopoietic cell injury [7, 8]. Nuclear factor erythroid 2-related factor 2 (Nrf2) is a transcription factor and a cellular sensor of oxidative stress [9]. In response to oxidative stress, Nrf2 dissociates from its cytoplasmic repressor kelch-like ECH-associated protein 1 (Keap1) and translocates into the nucleus, where it regulates the transcription of a wide variety of anti-oxidant genes, such as heme oxygenase-1(HO-1), NAD(P)H quinone oxidoreductases 1 (NQO1), and superoxide dismutase (SOD) [10, 11]. Moreover, Nrf2 is found to promote the survival of irradiated cells, including BM cells, through ROS scavenging [12]. These findings indicate that it is important to explore potent anti-oxidants to prevent ROS damage and protect HSCs from apoptosis caused by IR.

Astaxanthin (ATX), a natural anti-oxidant, is predominantly found in marine organisms and is the only known carotenoid that can be transported into the brain by transcytosis through the blood–brain barrier [13]. It exhibits powerful anti-oxidant activity compared to that of other carotenoids, such as canthaxanthin, lutein, lycopene, vitamin E, α-carotene, and β-carotene [14]. ATX can effectively scavenge intracellular free radicals and destroy peroxide chain reactions, thereby protecting cell and biological membranes from oxidative damage [15]. Previous studies have shown that ATX, owing to its biological source and superior anti-oxidant properties, exhibits numerous health benefits, including anti-tumor effects, anti-inflammatory actions, anti-diabetic effects, hepatoprotective effects, and immunomodulatory activity [16, 17]. Zhao and colleagues demonstrated that 30 days of ATX administration (1 mg/kg, 2 mg/kg, and 4 mg/kg) before irradiation protected against oxidative impairment and DNA damage in irradiated mice liver cells [18]. When referred to the hematopoietic system, ATX increased the counts of peripheral granulocytes and BM nucleated cells after mice received 8 Gy ^60^Co γ-rays, which illustrated preliminary that ATX may alleviate TBI-induced hematopoietic system injury. However, the anti-oxidant effect of ATX on TBI-induced HSC and HPC injury, and the underlying mechanisms have not been studied. Moreover, several studies have shown that ATX can attenuate apoptosis in many systemic diseases and cells [15, 19]. These studies suggest that application of ATX may alleviate hematopoietic cell apoptosis after TBI.

In this study, we explore the anti-oxidative and anti-apoptotic properties of ATX in TBI-induced hematopoietic system injury and the underlying molecular mechanisms. The results show that ATX improved TBI-induced skewed differentiation of peripheral blood cells and ameliorated BM suppression by accelerating hematopoietic self-renewal and regeneration. These findings were associated with the scavenging of ROS according to Nrf2-mediated increased activation of anti-oxidant proteins. Further study showed that the reduction of apoptosis in c-kit positive cells by regulating apoptotic-related proteins is also attributable to the radioprotective effects of ATX.

## Methods

### Reagents

Anti-mouse CD117 (c-kit)-APC (clone 2B8), anti-mouse Ly-6 A/EA (Sca-1)-PE/Cy7 (clone D7), biotin-conjugated anti-mouse CD4 (clone GK1.5), anti-mouse CD8 (clone 53–6.7), anti-mouse CD45R/B220 (cloneRA3-6B2), anti-mouse Ly6G/Gr-1 (clone RB6-8C5), anti-mouse CD11b (clone M1/70), anti-mouse Ter-119 (clone Ter-119), and APC-Cy7-conjugated streptavidin were obtained from eBioscience (San Diego, CA, USA). Anti-mouse CD45.1-FITC (clone A20, Ly5.1), anti-mouse CD45.2-PE (clone104, Ly5.2), anti-mouse Ly6G/Gr-1-PE/Cy7 (cloneRB6-8C5), anti-mouse CD45R/B220-PerCP (cloneRA3-6B2), anti-mouse CD11b-PE/Cy7 (cloneM1/70), anti-mouse CD3-APC (clone145-2C11), and streptavidin-PerCP (405213) were obtained from Biolegend (San Diego, CA, USA). Anti-mouse γH2AX and anti-mouse cleaved CASPASE 3 antibodies were obtained from Cell Signaling Technology (Danvers, MA, USA). Anti-mouse HO-1, anti-mouse NQO1, anti-mouse cytochrome C, anti-mouse β-ACTIN, anti-mouse NRF2, anti-mouse BAX, anti-mouse BAK, and anti-mouse BCL-XL antibodies were obtained from Abcam (Cambridge, UK). FITC-conjugated goat anti-rabbit/mouse antibodies were obtained from ZSGB-BIO (Beijing, China). Cytofix/Cytoperm buffer and Perm/Wash buffer were obtained from BD Pharmingen (San Diego, CA, USA). ATX was obtained from Aladdin Co. (Shanghai, China).

### Mice

Male C57BL/6 (CD45.2) mice were purchased from the Beijing HFK Bioscience Co. Ltd. (Beijing, China). Male C57BL/6 (CD45.1) mice were purchased from the Institute of Hematology and Blood Disease Hospital (PUMC, Tianjin, China). *Nrf2* ^*−/−*^ mice were generously provided by Dr. Thomas W. Kensler of the University of Pittsburgh, USA. All mice were approximately 6–8 weeks old (20–22 g) and housed under specific pathogen-free conditions at the Experimental Animal Centre of the Institute of Radiation Medicine of PUMC. All animal studies were approved by the Animal Care and Ethics Committee of the Institute of Radiation Medicine of PUMC (SYXK-2014-0002).

### TBI and ATX administration

Two batches of CD45.2 mice were respectively divided into (1) five groups: control group, TBI group, TBI + 25 mg/kg ATX group, TBI + 50 mg/kg ATX group, and TBI + 100 mg/kg ATX group; and (2) four groups: control group, ATX group, TBI group, TBI + ATX group (the ATX concentration was 50 mg/kg/day). *Nrf2* ^*−/−*^ mice were divided into three groups: control, TBI alone group, and TBI + ATX group. Each group had five mice. All mice that received ATX were administrated the compound by gavage 3 days before irradiation and 7 days after irradiation. Mice in all the TBI groups received 4 Gy γ-ray at a dose rate of 0.99 Gy/min. Control mice were sham-irradiated.

### Weight and organ index

The mean body weights of the groups were plotted to determine the weight gain or loss in the control and test groups. Then the mice were sacrificed and the organs were excised and weighed. Organ weights were recorded, and indices (in g/g) were calculated by the ratio of the wet weights of the individual organs to the whole body weights.

### Peripheral blood cell and bone marrow cell counts

Blood was obtained from the mice via the orbital sinus and was collected in micropipettes coated with the ethylenediaminetetraacetic acid (EDTA.K3). The cell counts included white blood cells (WBCs), percentage of lymphocytes (LY%), and percentage of neutrophil granulocytes (NE%). Bone marrow cells were flushed from both the tibias and femurs with sterile phosphate-buffered saline (PBS), and the cell numbers were counted using a Celltac E hemocytometer (Nihon Kohden, Japan).

### Flow cytometry analysis

For B cell, T cell, and myeloid cell analysis in peripheral blood, 50 μl peripheral blood was first incubated with B220, CD3, CD11b, and Gr1 at room temperature, and then the red blood cells were removed with BD FACS™ Lysing Solution. For HPC and HSC analysis, bone marrow cells were isolated as described above. They were then filtered and counted prior to staining with antibodies. Bone marrow cells (5 × 10^6^) were incubated with biotin-labeled antibodies specific for murine Ter119, B220, Gr1, CD11b, CD4, and CD8, and were then stained with streptavidin, c-kit, and sca1. For HO-1, NQO1, γH2AX, and cytosol cytochrome C (cyt C) analysis, bone marrow cells (5 × 10^6^) were stained with c-kit antibody, fixed, and permeabilized using BD Cytofix/Cytoperm buffer according to the manufacturer’s protocol, and finally stained with respective antibodies and FITC-conjugated secondary antibodies. Data acquisition was performed using a BD Accuri C6 and analyzed using BD Accuri C6 software.

### Competitive repopulation assay

Bone marrow cells (1 × 10^6^) from C57BL/6 (CD45.2) mice after the various treatments and 1 × 10^6^ bone marrow cells from C57BL/6 (CD45.1/45.2) mice were mixed and transplanted into lethally irradiated C57BL/6 mice (CD45.1). The percentage of donor-derived (CD45.2) cells in the recipients’ peripheral blood was examined 2 months after transplantation.

### Colony of granulocyte macrophage cells (CFU-GM) assay

Bone marrow cells (1 × 10^4^) from the control groups and 1 × 10^5^ bone marrow cells from the TBI groups were cultured in M3534 methylcellulose medium (Stem Cell Technologies) for 5 days. The colonies of CFU-GM with more than 30 cells were counted according to the instructions. The results are expressed as the numbers of CFU-GM per 10^5^ bone marrow cells.

### Analysis of intracellular ROS levels

Bone marrow cells (5 × 10^6^) from wild mice or *Nrf2* ^*−/−*^ mice were first stained with c-kit antibody, and were then incubated with 2′, 7′-dichlorodihydrofluorescein diacetate (DCFDA; Beyotime Biotechnology, Nanjing, China; 10μM), MitoSOX (Life Technologies, Grand Island, NY, USA; 10μM); and dihydroethidium (DHE; Beyotime Biotechnology; 5μM) for 20 min, 30 min, and 10 min, respectively, at 37 °C in a water bath according to the manufacturer’s protocol. The levels of intracellular ROS in c-kit-positive cells were analyzed by measuring the mean fluorescence intensity (MFI) of DCFDA, DHE, and oxidized MitoSOX using a flow cytometer.

### Analysis of SOD2, CAT, and GPX1 activity

To isolate c-kit-positive cells, bone marrow cells were stained with CD117 microbeads (Miltenyi Biotec, Teterow, Germany) for 20 min at 2–8 °C, and the c-kit-positive cells were collected according to the manufacturer’s protocol. SOD2, CAT, and GPX1 enzymatic activities in c-kit-positive cells were analyzed using SOD, CAT, and GPX1 assay kits (Beyotime Institute of Biotechnology, Jiangsu, China), respectively, following the manufacturer’s instructions.

### Immunofluorescence staining of NRF2

c-kit-positive cells were sorted as described above, and fixed in 4% paraformaldehyde for 10 min at room temperature. Cells were permeabilized in 0.2% Triton-X-100/PBS and blocked with 5% bovine serum albumin (BSA) for 1 h at room temperature, and then incubated with anti-NRF2 antibody (1:300; overnight at 4 °C). Cells were washed three times with PBS for 5 min, and incubated with FITC-conjugated goat anti-rabbit IgG (1:300) antibody in the dark for 1 h at room temperature. Finally, a fluorescent sealing liquid solution (ZSGB-BIO, Beijing, China) containing 4′,6-diamidino-2-phenylindole (DAPI) was added to stain the nuclei. Fluorescence images were visualized with a fluorescence microscope.

### Apoptosis assay

Bone marrow cells (5 × 10^6^) were first incubated with a c-kit antibody, and cell apoptosis was then evaluated using an Annexin V-FITC Apoptosis Detection Kit (BD Biosciences, San Jose, CA, USA) followed by flow cytometry analysis according to the manufacturer’s protocol. Finally, samples were evaluated by BD Accuri C6 and analyzed using BD Accuri C6 software.

### Analysis of cytochrome C release from mitochondria in c-kit-positive cells

c-kit-positive cells were sorted as described above. The cytochrome C release from mitochondria in the c-kit-positive cells was determined by analysis of the MFI by flow cytometry according to the method described previously [20].

### Western blotting analysis

c-kit-positive cells were collected as described above and then lysed using RIPA reagent (Boster Biological, Wuhan, China) supplemented with a protease inhibitor cocktail (Sigma, St. Louis, MO, USA) and phenylmethylsulfonyl fluoride (PMSF; Sigma). Proteins were separated by 12% SDS-polyacrylamide gel electrophoresis, transferred to nitrocellulose membranes (Sigma), and detected using specific antibodies as follows: β-ACTIN antibody (1:1000), NRF2 antibody (1:1000), BAX antibody (1:1000), BAK antibody (1:1000), BCL-XL antibody (1:1000), and cleaved CASPASE 3 antibody (1:1000). Blots were developed using Molecular Imager Chemi Doc™ XRS+ with Image Lab™ Software (Bio-Rad, Richmond, CA, USA).

### Statistical analysis

Statistical analysis was carried out using GraphPad Prism 5 software with a *t* test and Welch’s correction. Differences were considered significant at *P* < 0.05.

## Results

### ATX rescues the loss of body weight and the changes in organ index caused by TBI in mice

**Fig. 1:**
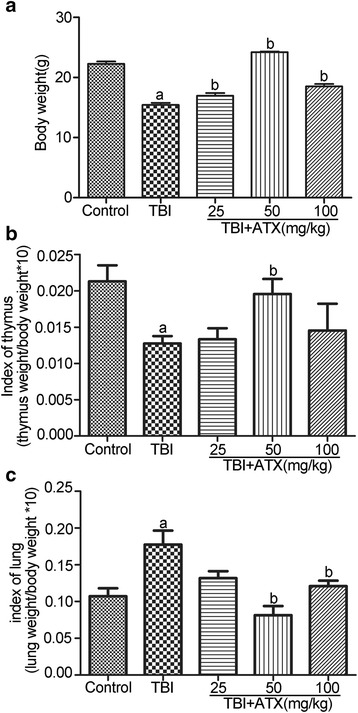
Astaxanthin (*ATX*) rescues the body weight loss and the organ index changes caused by total body irradiation (*TBI*) in mice. Mice were randomly divided into five groups and were administered either DMSO or different concentrations of ATX by gavage for 3 days before exposure to 4 Gy TBI and then continuously for 7 days after irradiation. Control mice were sham-irradiated. **a** Bar graph showing the average body weight of each group. **b** Bar graph showing the thymus index. **c** Bar graph showing the lung index. The data are presented as the mean ± SEM (*N* = 5). ^a^ *P* < 0.05 vs control; ^b^ *P* < 0.05 vs TBI

Compared to the control group, the body weight of the TBI group had decreased significantly when mice were sacrificed 12 days after 4 Gy irradiation. However, the body weight of mice that received 25-100 mg/kg ATX was significantly increased compared to that of the TBI groups (Fig. 1a). In addition, compared with TBI group, the index of thymus of the 50 mg/kg group was significantly rescued, and the indices of lung in TBI + ATX groups showed a significant improvement (Fig. 1b and c). Our results suggested that 50 mg/kg ATX has effective radioprotective effects on TBI-induced injury in mice.

### ATX can improve radiation-induced skewed differentiation of peripheral blood cells

**Fig. 2:**
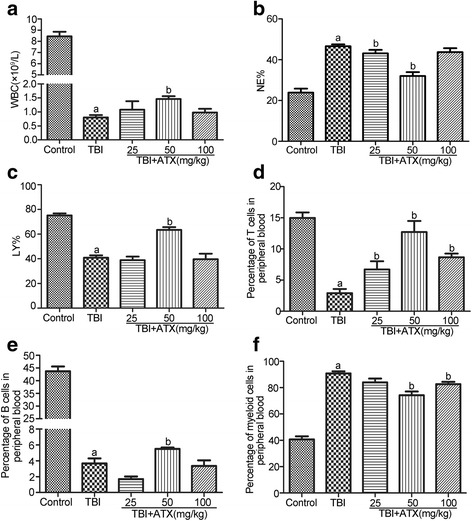
Astaxanthin (*ATX*) attenuates total body irradiation (*TBI*)-induced multilineage differentiation disorders and maintains hematopoietic homeostasis. Mice were sham-irradiated as a control or irradiated with 4 Gy TBI after receiving DMSO or various concentrations of ATX in a manner similar to that illustrated in Fig. 1. **a** Bar graph showing the numbers of white blood cells (*WBC*). **b** Bar graph showing the percentage of neutrophilic granulocytes (*NE%*) and (**c**) lymphocytes (*LY%*). The percentage of T cells (**d**), B cells (**e**), and myeloid cells (**f**) in peripheral blood detected by flow cytometry. The data are presented as the mean ± SEM (*N* = 5). ^a^ *P* < 0.05 vs control; ^b^ *P* < 0.05 vs TBI

HSCs not only have the capacity for self-renewal but can also differentiate into different blood cell lineages which must be balanced to prevent lineage skewing [1]. To assess the possible role of ATX in protecting the hematopoietic system against radiation exposure, we examined the abundance of different types of hematopoietic cells in peripheral blood samples collected from the five groups described above using a hemocytometer and flow cytometry. As shown in Fig. 2, TBI-treated mice exhibited a substantial reduction in WBCs and percentage of lymphocyte cells (including T cells and B cells), while the percentage of neutrophils (NE%) and myeloid cells increased. Treatment with 50 mg/kg ATX notably rescued impaired differentiation of peripheral blood cells in the mice exposed to 4 Gy radiation, which demonstrated that ATX can effectively restore multilineage differentiation disorders and maintain hematopoietic homeostasis.

### ATX treatment attenuates TBI-induced bone marrow cell reduction in vivo

**Fig. 3:**
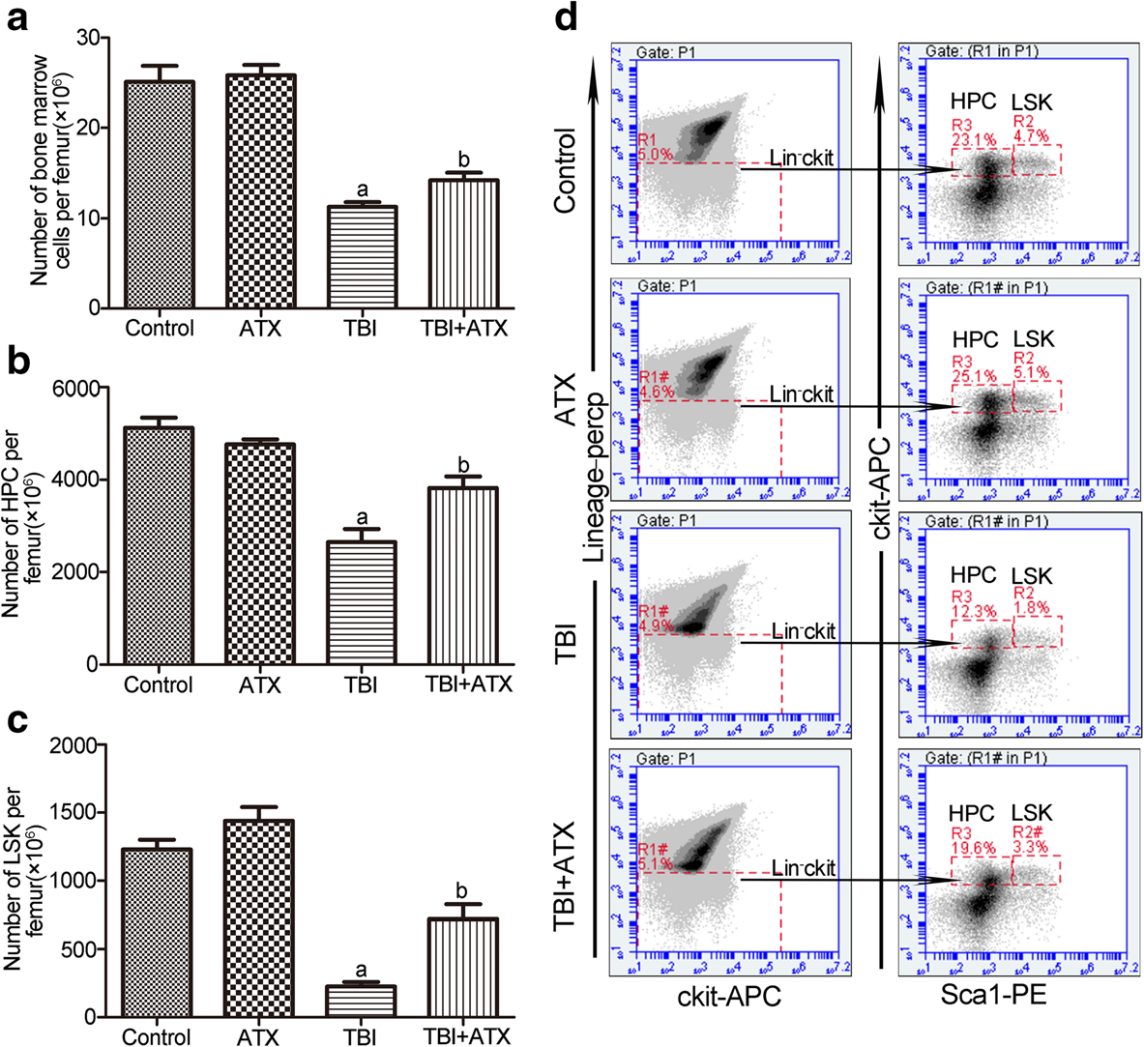
Astaxanthin (*ATX*) treatment attenuates total body irradiation (*TBI*)-induced bone marrow cell reduction in vivo. Mice were randomly divided into four groups and were administered DMSO or 50 mg/kg ATX by gavage for 3 days before exposure to 4 Gy TBI and then continuously for 7 days after irradiation. Control/ATX mice were sham-irradiated. All mice were sacrificed at 12 days after exposure to TBI. The numbers and frequencies of hematopoietic progenitor cells (*HPCs*) and LSK cells in bone marrow were then analyzed by flow cytometry. **a** Bar graph showing the numbers of bone marrow cells per femur. **b** The numbers of HPCs (Lineage^−^Sca1^−^c-kit^+^ bone marrow cells) per femur. **c** The numbers of LSKs (Lineage^−^Sca1^+^c-kit^+^ bone marrow cells) per femur. **d** Representative FACS plots show the percentages of HPC and LSK. The data are presented as the mean ± SEM (*N* = 5). ^a^ *P* < 0.05 vs control; ^b^ *P* < 0.05 vs TBI

To determine whether ATX treatment could promote recovery of HSCs in vivo, we irradiated C57BL/6 mice with 4 Gy and then treated them with 50 mg/kg ATX or DMSO via oral gavage from day −3 to day +7. At day +12 following TBI, ATX-treated mice had increased numbers of BM cells, LSK cells (Lin^−^Sca-l^+^c-kit^+^), and HPCs (Lin^−^Sca-l^−^c-kit^+^), as well as increased percentages of LSK cells and HPCs compared to vehicle-treated mice (Fig. 3). These results suggested that ATX significantly increased the recovery of BM HPCs/LSK cells after TBI.

### Systemic administration of ATX improves HSC regeneration in vivo

**Fig. 4:**
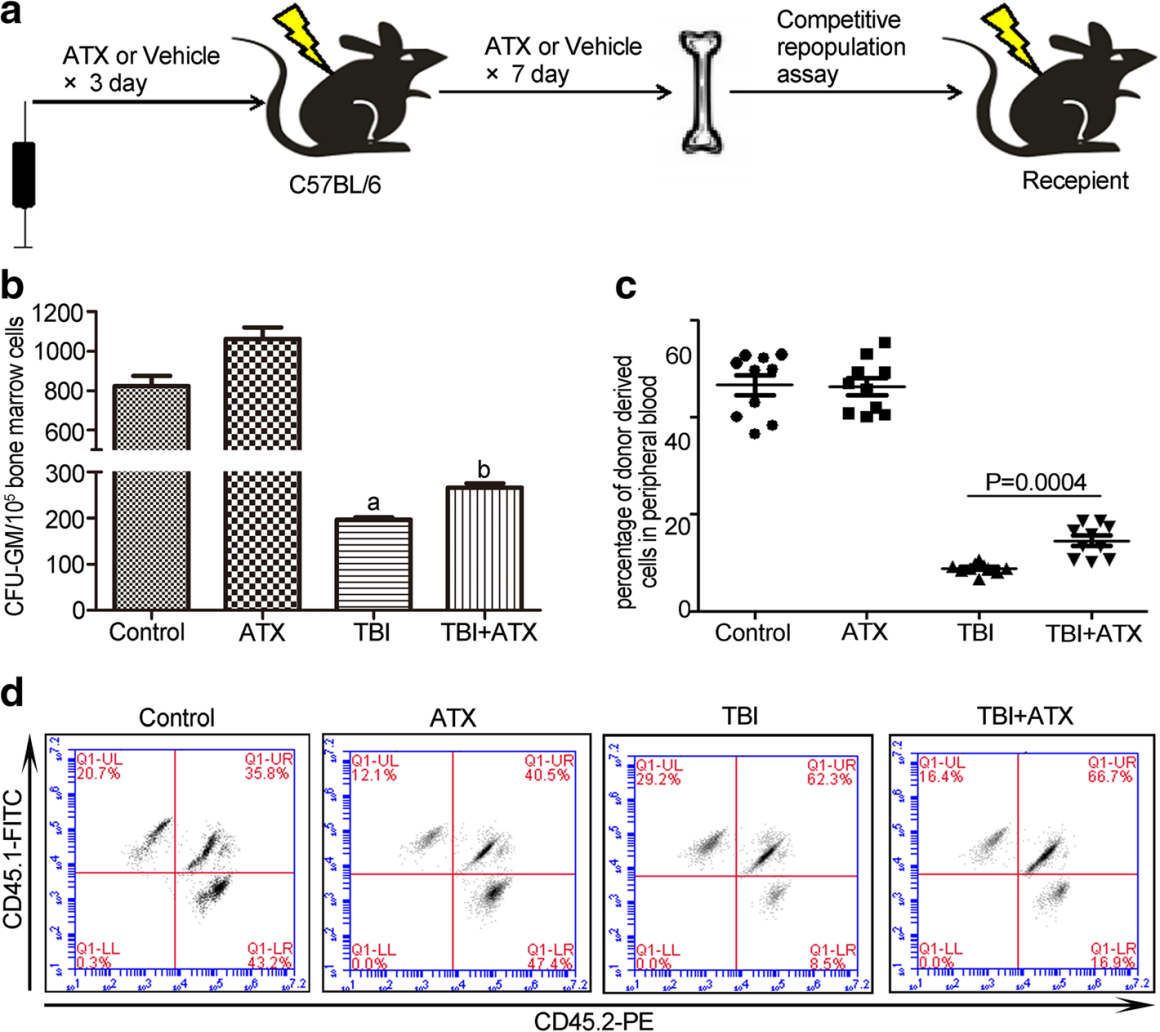
Astaxanthin (*ATX*) reduces total body irradiation (*TBI*)-induced suppression of BM cell clonogenic function and induces HSC reconstitution in vivo. **a** Schematic diagram shows the recipients were transplanted with BM cells from the various treated groups. **b** Bar graph showing the number of CFU-GM per 10^5^ BM cells. **c** The scatter plot shows the percentage of donor-derived cells in peripheral blood cells 2 months after transplantation. **d** Representative results of donor cell engraftment. The data are presented as the mean ± SEM (*N* = 6 in **b** and *N* = 10 in **c**). ^a^ *P* < 0.05 vs control; ^b^ *P* < 0.05 vs TBI

To determine if treatment with ATX could rescue the reconstituted function of the HSC pool in irradiated mice, we performed CFU-GM assays and competitive BM transplant experiments. The results showed that, compared to the control group, the number of CFU-GM in the TBI group decreased significantly, and ATX treatment increased the CFU-GM number in 4 Gy irradiated mice (Fig. 4b). In the competitive BM transplant experiments, lethally irradiated (CD45.1^+^) recipient mice were injected with 1 × 10^6^ donor (CD45.2^+^) BM cells from the mice in the control group, control + ATX group, 4 Gy group, and 4 Gy + ATX group. At 2 month post-transplantation, we measured donor-derived cells (CD45.2^+^) in the peripheral blood of recipient mice by FACS. Chimerism of donor cells from 4 Gy irradiated mice was significantly lower than that of non-irradiated mice, while the 4 Gy + ATX treatment transplanted group displayed a corresponding increase in donor cell chimerism (Fig. 4c). These results suggested that ATX promotes the recovery of HSC regeneration potential in 4 Gy irradiated mice.

### ATX scavenges TBI-induced ROS in BM c-kit-positive cells

**Fig. 5:**
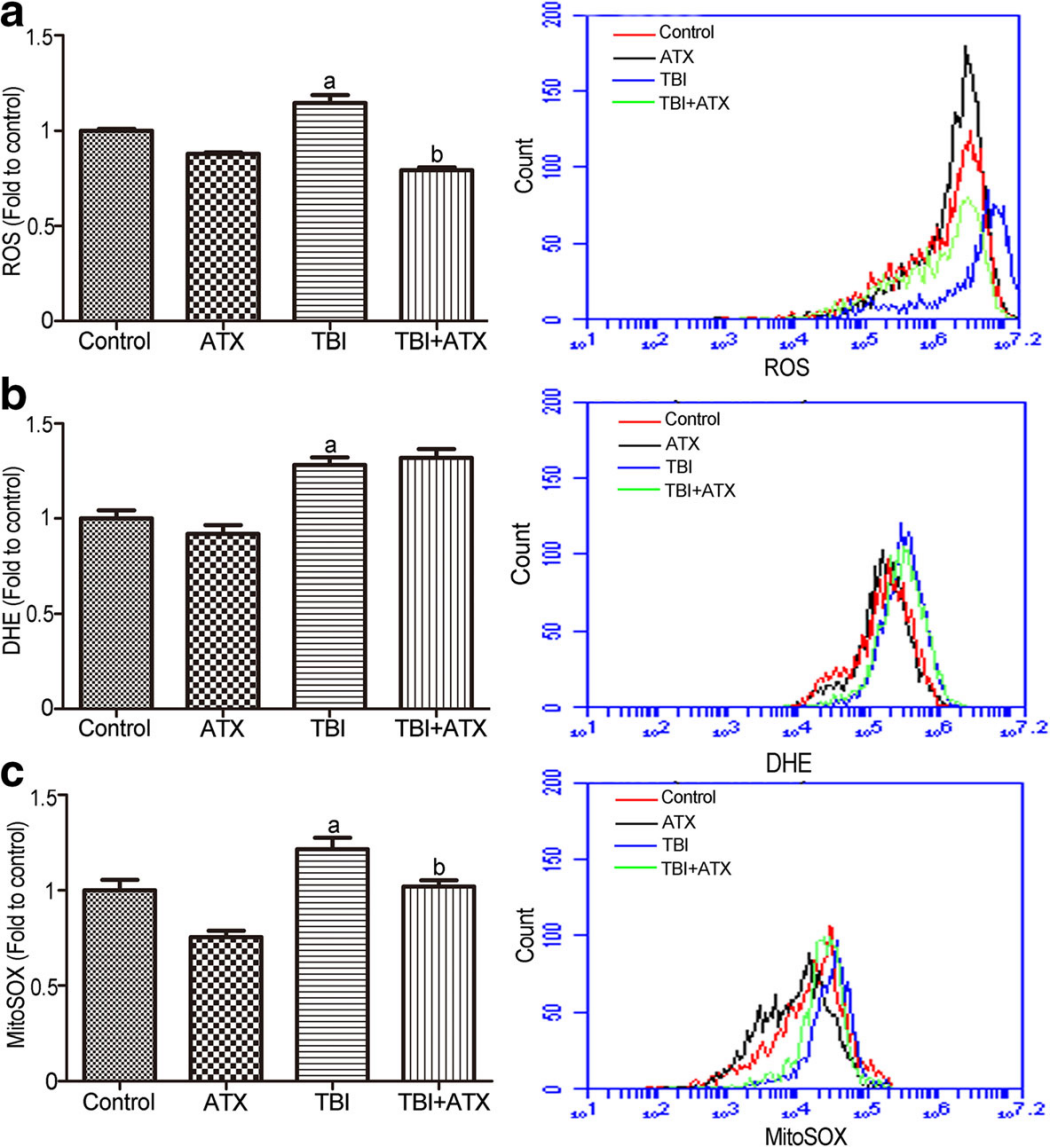
Astaxanthin (*ATX*) treatment inhibits total body irradiation (*TBI*)-induced reactive oxygen species (*ROS*) generation in BM c-kit-positive cells. **a** The levels of ROS detected by the DCFDA MFI and the representative analysis of ROS levels by flow cytometry. **b** The levels of ROS detected by the DHE MFI and the representative analysis of DHE levels by flow cytometry. **c** The levels of ROS in mitochondria detected by the MitoSOX MFI and the representative analysis of MitoSOX levels by flow cytometry. The data are presented as the mean ± SEM (*N* = 5). ^a^ *P* < 0.05 vs control; ^b^ *P* < 0.05 vs TBI

Exposure to irradiation can increase ROS generation which may cause BM suppression [21]. ATX is increasingly used in the nutritional and healthcare industry owing to its superior anti-oxidant properties [22]. Therefore, to determine whether ATX can attenuate TBI-induced BM suppression by eliminating ROS, we measured the ROS production in BM c-kit-positive cells 12 days after TBI by flow cytometry. As shown in Fig. 5, the generation of ROS in c-kit-positive cells, which was analyzed by DCFDA, DHE, and MitoSOX staining, was significantly elevated after TBI compared to non-irradiated mice. ATX treatment markedly attenuated the elevation of ROS production detected by DCFDA and MitoSOX, but not DHE, suggesting that ATX can effectively scavenge TBI-induced ROS production, especially those from mitochondria, while the scavenging effect of ATX on superoxide anion free radicals was not as strong. These results indicated that ATX acts as an inhibitor (anti-oxidant) of TBI-mediated ROS generation.

### ATX upregulates expression of NRF2 and target anti-oxidant proteins

**Fig. 6:**
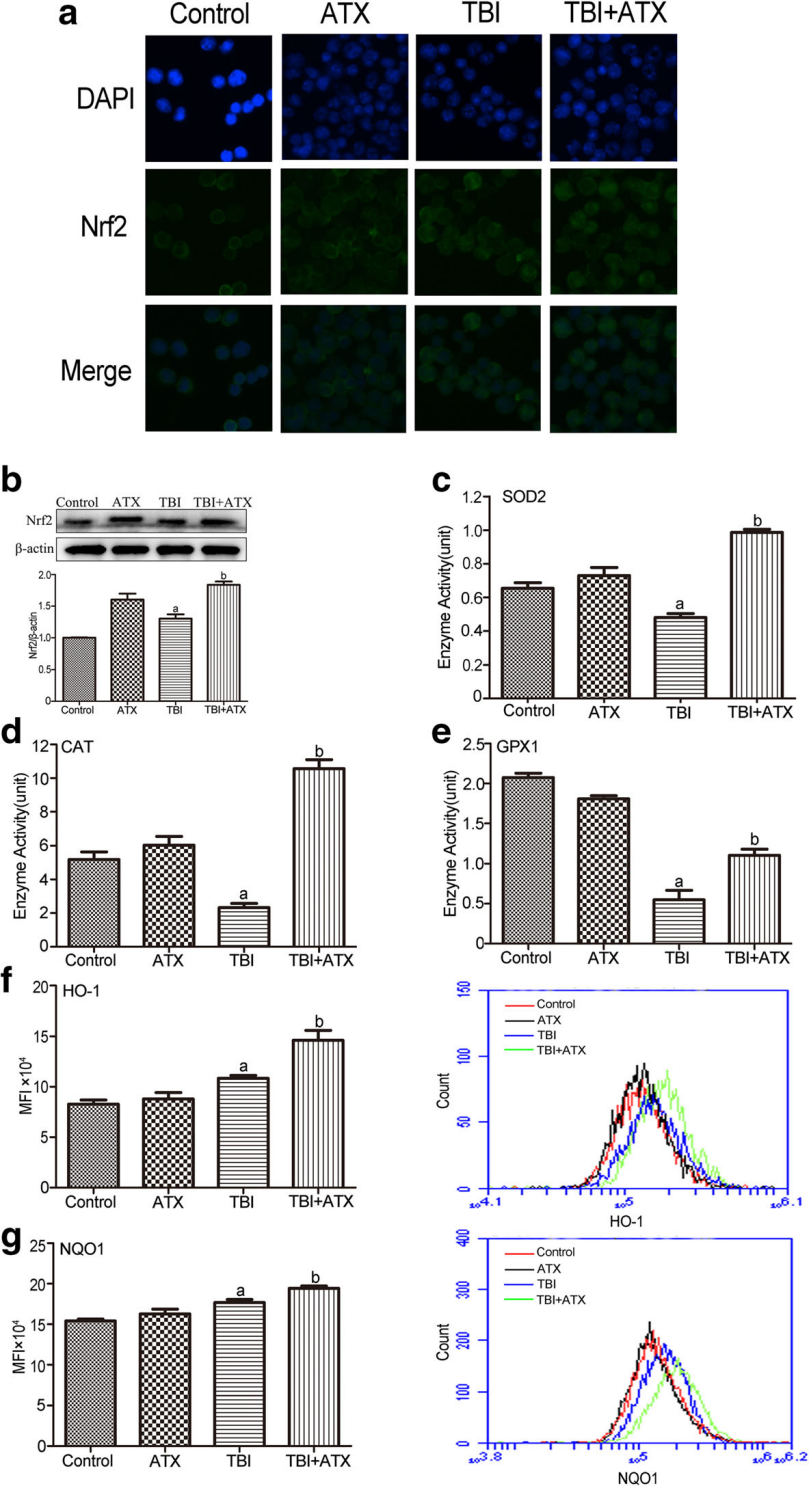
Astaxanthin (*ATX*) scavenges total body irradiation (*TBI*)-induced excessive ROS by upregulating NRF2 and its downstream anti-oxidant proteins. **a** The NRF2 protein level in c-kit-positive cells was determined using the immunofluorescence method. **b** Expression of NRF2 protein was analyzed by Western blotting. The enzyme activities of **c** SOD2, **d** CAT, and **e** GPX1 were analyzed by respective assay kit. The levels of intracellular **f** HO-1 and **g** NQO1 expression was analyzed by flow cytometry. The data are presented as the mean ± SEM (*N* = 3 in **b**–**e** and *N* = 5 in **f**, **g**). ^a^ *P* < 0.05 vs control; ^b^ *P* < 0.05 vs TBI. *MFI* mean fluorescence intensity

Nrf2 is the central regulator of endogenous anti-oxidant defense, the activation of which plays an important role in regulating cellular redox homeostasis [10]. Western blotting analysis and immunofluorescence assays showed that TBI significantly upregulated NRF2 protein expression and promoted nuclear translocation; these alterations were enhanced by ATX (Fig. 6a and b). Furthermore, ATX also increased the expression of NRF2-targeted proteins HO-1 and NQO1 (Fig. 6f and g). It has been shown that ATX attenuated intracellular ROS production in various pathological conditions by restoring the anti-oxidant enzyme activities of SOD2, CAT, and GPX1 [23]. We found that a decreased enzyme activity for SOD2, CAT, and GPX1 was observed in irradiated c-kit-positive cells, which were also enhanced by ATX (Figs. 6c–e). These findings indicated that ATX suppresses ROS generation by activating NRF2 and upregulating expression of related anti-oxidant enzymes and proteins.

### ATX decreases ROS levels in *Nrf2*^*−/−*^ c-kit-positive cells with TBI

**Fig. 7:**
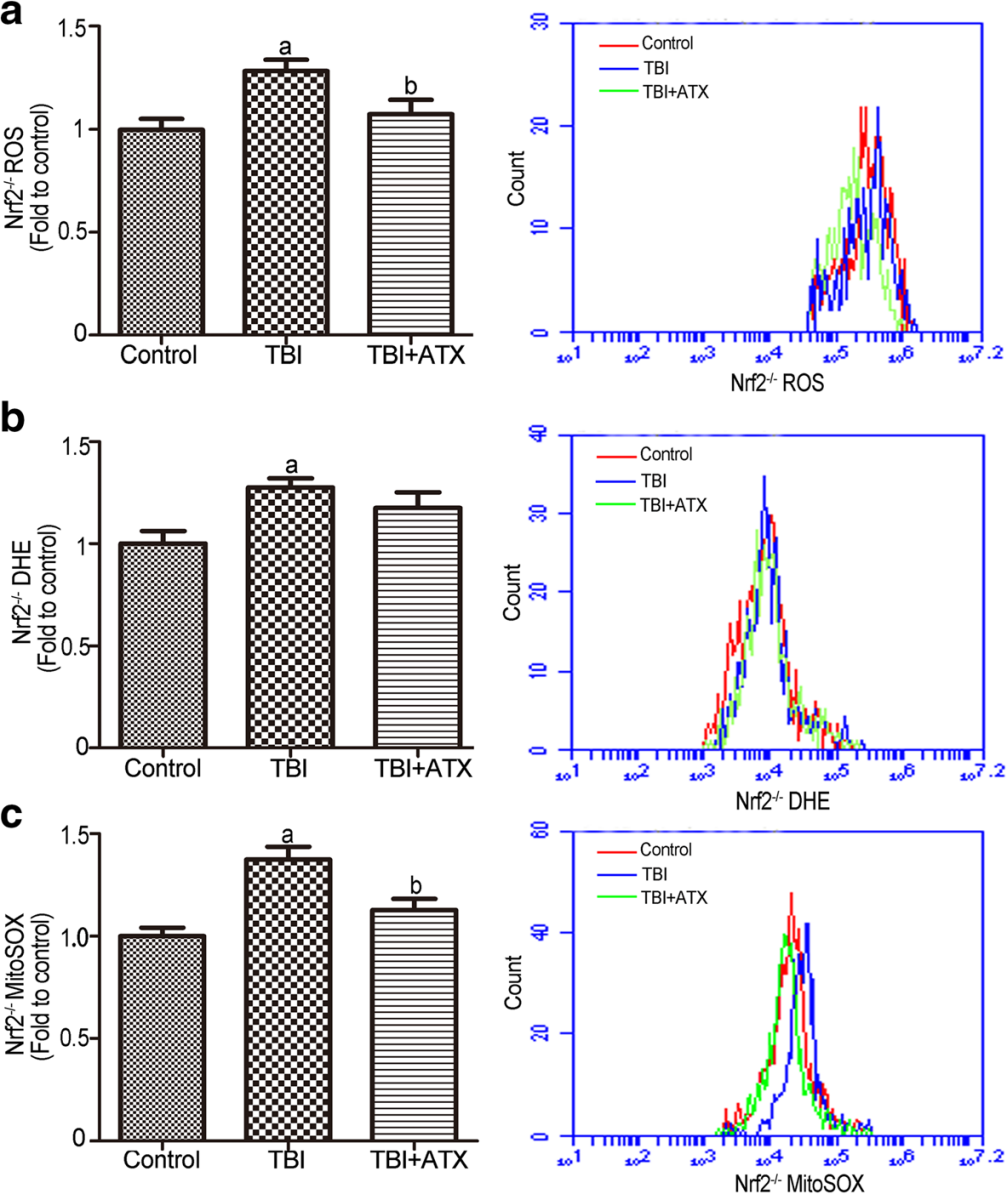
Astaxanthin (*ATX*) alleviates total body irradiation (*TBI*)-induced *Nrf2* ^*−/−*^ reactive oxygen species (*ROS*) generation in c-kit-positive cells. **a** The levels of ROS detected by the DCF MFI and the representative analysis of ROS levels by flow cytometry. **b** The levels of ROS detected by the DHE MFI and the representative analysis of DHE levels by flow cytometry. **c** The levels of ROS in mitochondria detected by the MitoSOX MFI and the representative analysis of MitoSOX levels by flow cytometry. The data are presented as the mean ± SEM (*N* = 5). ^a^ *P* < 0.05 vs control; ^b^ *P* < 0.05 vs TBI

To confirm the important role of Nrf2 in ATX-mediated ROS scavenging, further experiments were conducted to explore whether ATX decreased ROS levels in c-kit-positive cells of irradiated *Nrf2* ^*−/−*^ mice. As shown in Fig. 7, ATX indeed decreased the ROS levels in c-kit-positive cells of irradiated *Nrf2* ^*−/−*^ mice as evidenced by DCFDA and MitoSOX, but not DHE. These results suggested that ATX mediated ROS scavenging in irradiated c-kit-positive cells, partly by activation of the Nrf2-dependent pathway.

### ATX inhibits TBI-induced DNA DSBs and apoptosis in c-kit-positive cells

**Fig. 8:**
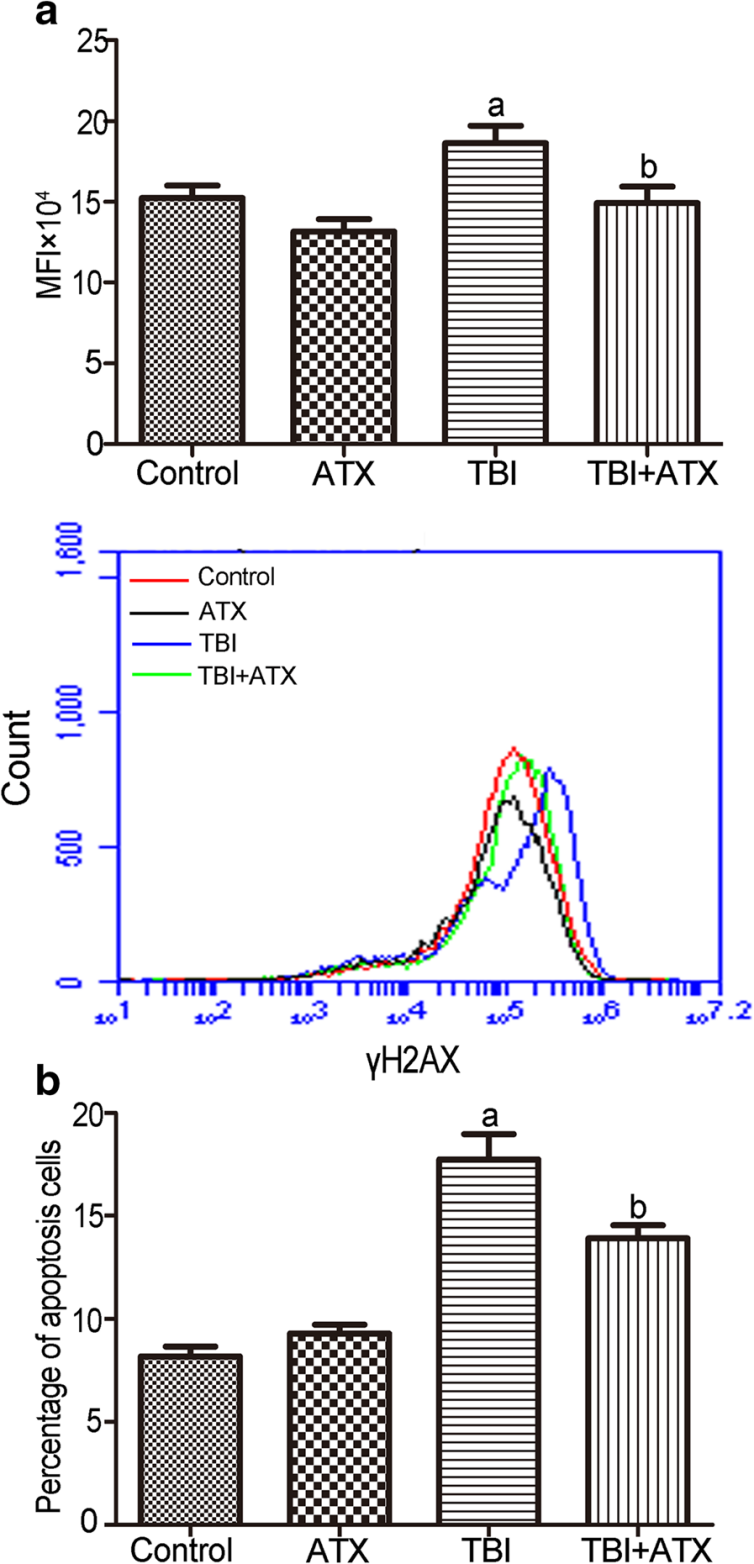
Astaxanthin (*ATX*) reduces total body irradiation (*TBI*)-induced DNA DSBs and apoptosis in c-kit-positive cells. **a** Representative analysis of γH2AX expression in c-kit-positive cells by flow cytometry. **b** Bar graph showing the percentage of apoptosis in c-kit-positive cells. The data are presented as the mean ± SEM (*N* = 5). ^a^ *P* < 0.05 vs control; ^b^ *P* < 0.05 vs TBI. *MFI* mean fluorescence intensity

TBI may induce DNA DSBs that lead to duplication and transcriptional errors, cell apoptosis, or dysfunctional cell growth, which finally results in hematopoietic diseases [24]. We next performed flow cytometric analysis of Annexin/PI staining and γH2AX staining to assess the effects of ATX on c-kit-positive cell survival and DNA damage. As shown in Fig. 8a, γH2AX staining in irradiated c-kit-positive cells was significantly increased after TBI. Conversely, treatment with ATX substantially reduced TBI-induced DNA damage in c-kit-positive cells, as shown by the reduced γH2AX level. Notably, the γH2AX level of the irradiated c-kit-positive cells was almost at normal levels after ATX treatment, indicating that ATX treatment can ameliorate TBI-induced DSBs in c-kit-positive cells. IR-induced cell death in HSCs predominantly occurs by apoptosis but not by necrosis [5]; thus, apoptosis was quantified (Fig. 8b). The percentage of apoptotic cells increased significantly after irradiation compared with non-irradiated cells, but ATX administration markedly reduced this trend. These findings showed that ATX could prevent TBI-induced apoptosis of HSCs/HPCs in vivo. Taken together, these findings suggested that ATX significantly alleviated hematopoietic failure by attenuating TBI-induced DNA damage and apoptosis in c-kit-positive cells.

### ATX alleviates TBI-induced BM suppression via modulation of the apoptotic pathway

**Fig. 9:**
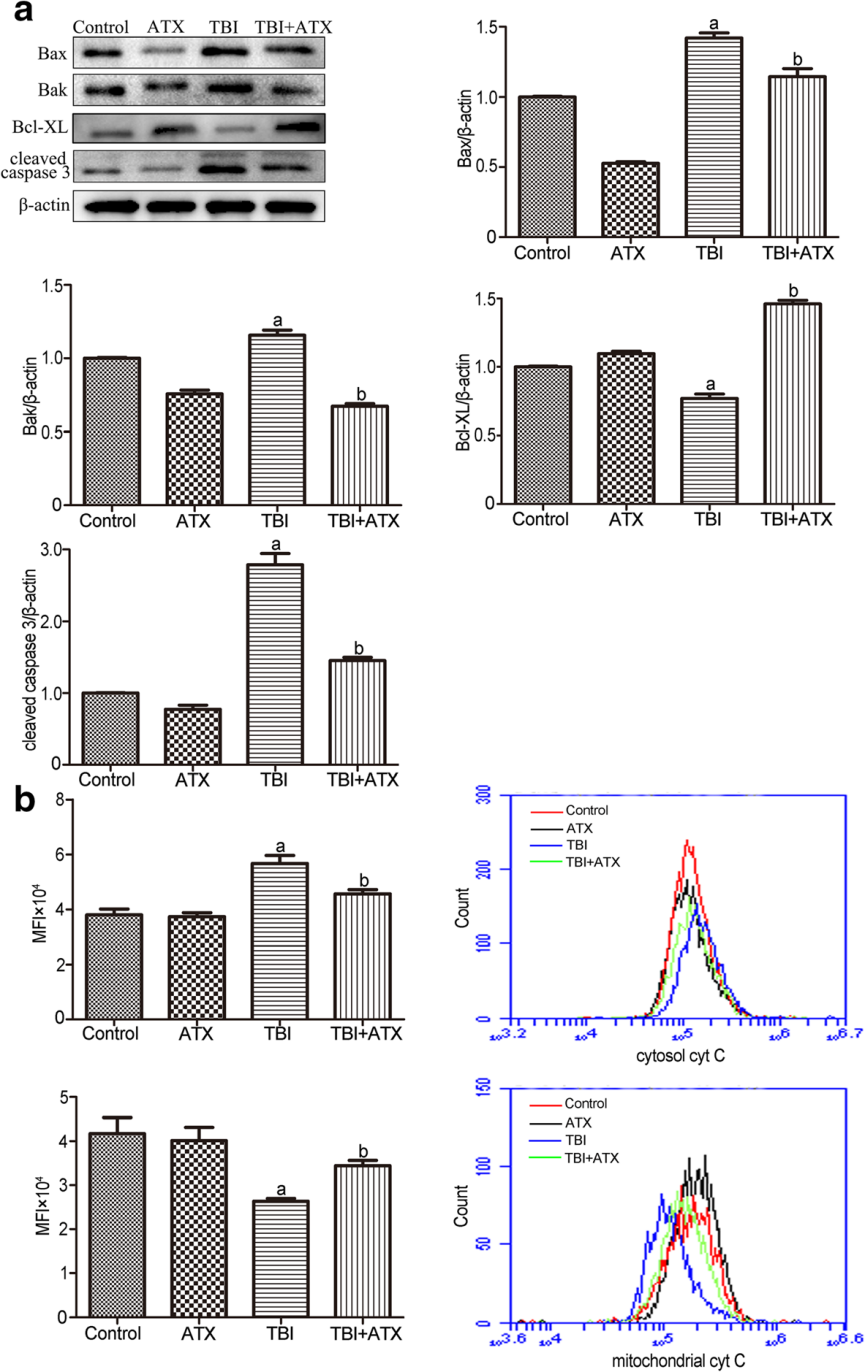
Astaxanthin (*ATX*) protects the hematopoietic system from total body irradiation (*TBI*) by regulating the apoptotic pathway. **a** The protein expression of BAX, BAK, BCL-XL, and cleaved CASPASE 3 in c-kit-positive cells was analyzed by Western blotting. **b** Representative analysis of cytochrome C (*cyt C*) release by flow cytometry. The data are presented as the mean ± SEM (*N* = 3 in **a** and *N* = 5 in **b**). ^a^ *P* < 0.05 vs control; ^b^ *P* < 0.05 vs TBI. *MFI* mean fluorescence intensity

Cellular apoptosis is mediated by the balance of pro- and anti-apoptotic proteins. Specifically, the apoptotic pathway is regulated by early translocation of anti-apoptotic (Bcl-xl) and pro-apoptotic (Bak and Bax) proteins to or from the mitochondria [25]. Therefore, we assessed the protein levels of BCL-XL, BAK, and BAX during apoptosis in c-kit-positive cells after TBI. The expression of BCL-XL significantly decreased in the TBI group, while the pro-apoptotic proteins showed the opposite trend. However, ATX application significantly ameliorated the increased expression of BAK and BAX and upregulated the protein expression of BCL-XL compared with the TBI groups (Fig. 9a). Next, we detected the cytochrome C level both in cytosol and the mitochondria. Results showed that TBI induced cytochrome C release from the mitochondria to cytosol, while ATX significantly blocked the efflux of cytochrome C from the mitochondria (Fig. 9b). Compared to the control group, an upregulated expression of cleaved CASPASE 3 was observed in mice with TBI. ATX downregulated the expression of cleaved CASPASE 3 in irradiated c-kit-positive cells. These data suggested that ATX could reduce apoptosis in c-kit-positive cells following TBI by regulating the apoptotic pathway.

### ATX improves the survival of lethally irradiated mice

**Fig. 10:**
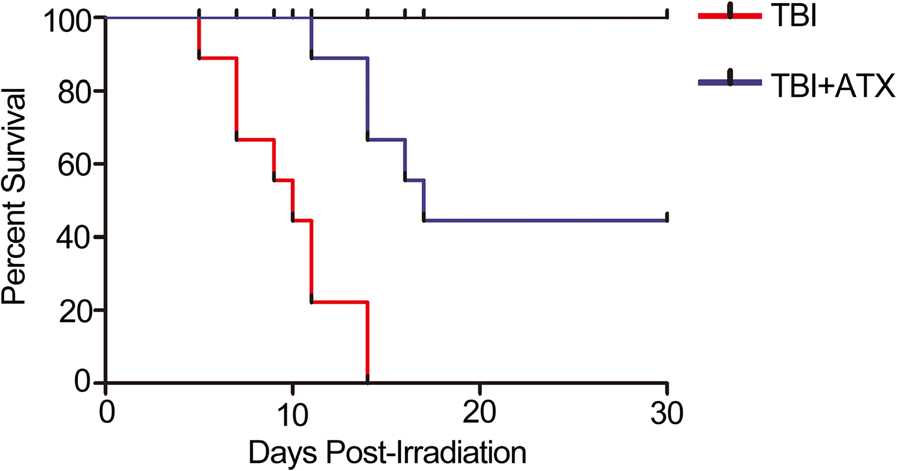
Astaxanthin (*ATX*) administration increases survival. Mice were treated with DMSO or ATX (50 mg/kg) by gavage for 3 days before exposure to a lethal dose (7.2 Gy) of total body irradiation (*TBI*) and then continuously for 7 days after TBI. Kaplan–Meier analysis of mouse survival after exposure to the lethal dose of TBI (*N* = 19 mice/group)

In this study, to test whether ATX can increase the survival of mice following TBI, mice were administered 50 mg/kg ATX or DMSO from day −3 to day +7 following lethal doses of TBI (7.2 Gy). The survival rate was monitored for 30 days after TBI. None of the control mice survived beyond 15 days after the lethal irradiation. In contrast, forty-four percent of the ATX-treated mice remained alive through day +30 (Fig. 10). These results suggested that administration of ATX increased the survival of mice following a lethal dose of TBI.

## Discussion

ATX is extensively used as food colorant and is approved by the United States Food and Drug Administration (FDA) [26], and is a healthcare product to prevent several human diseases owing to its natural source and superior anti-oxidant activity [27, 28]. However, the therapeutic potential of ATX as a radiation protectant or mitigator has not been investigated. In the present study, we examined whether ATX can attenuate TBI-induced hematopoietic system injury. Consistent with previous studies, our results showed that 4 Gy TBI can induce myelosuppression mainly due to the generation of oxidative stress and the apoptosis of HSCs and HPCs [29].

Under our experimental conditions, 50 mg/kg ATX exhibited a greater protective activity than that from 25 mg/kg or 100 mg/kg ATX. These data suggested that ATX at low-doses was unable to efficiently protect mice against TBI-induced injury, while ATX, one kind of carotenoid, might cause a decrease in anti-oxidant capacity, and even the promotion of the oxidation reaction [30]. There was no significant difference in ROS level between TBI and TBI + 100 mg/kg ATX (data not shown), indicating a similar anti-oxidant capacity in these two groups. Similar observations with other protective agents, such as 3,3′-diindolylmethane, ascorbic acid, and Xuebijing, were also obtained in previous studies by us and other laboratories [31–35]. ATX at 50 mg/kg ameliorated TBI-induced myelosuppression, increased the numbers of HPCs, and increased self-renewal and differentiation ability in our present study.

In our experiment, ATX rescued body weight and ROS expression to a normal level 12 days after exposure to TBI; however, the hematopoietic system was still under recovery and residual HSCs reconstituted the hematopoietic system. Therefore, it is impossible for hematopoietic makers and bone marrow cells to be rescued to normal levels. Peripheral blood cell and bone marrow cell counts can recover 2 months after exposure to a moderate dose TBI while, because of persistent activation of ROS, TBI causes bone marrow cell senescence and long-term damage manifested as HSC pool exhaustion and severe damage to the reconstructive ability of bone marrow. Further studies are needed to explore the effect of ATX on TBI-induced long-term hematopoietic injury.

**Fig. 11:**
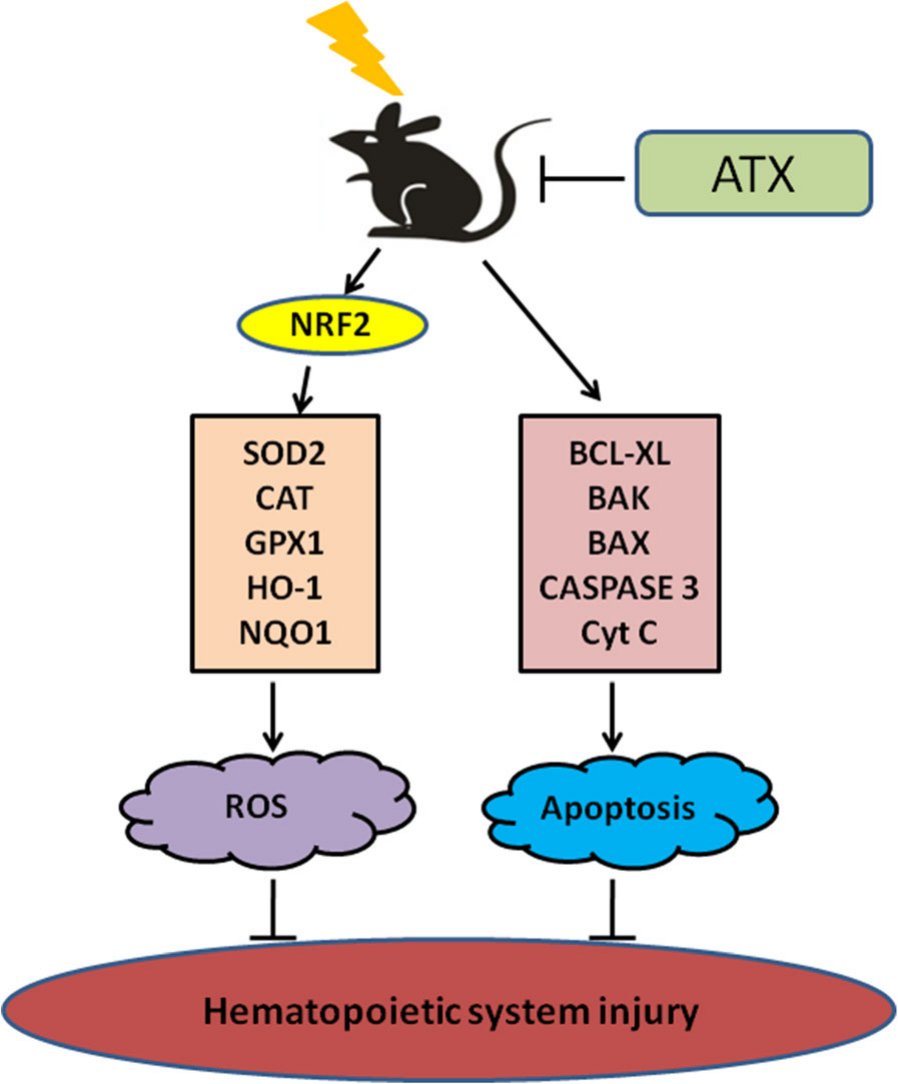
A Summary on the radioprotective mechanism of astaxanthin (*ATX*). *ROS* reactive oxygen species

It has been well established that TBI causes radiolysis of intracellular water molecules, leading to increased production of ROS [36]. As shown in our study, ATX markedly mitigated ROS production detected by DCFDA and MitoSOX, but not DHE, suggesting that ATX can scavenge ROS produced from mitochondria, but that it has no scavenging effect on superoxide anion free radicals. No change in SOD1 (which is found almost exclusively in intracellular cytoplasmic spaces) enzyme activity was observed in irradiated c-kit-positive cells treated with ATX (data not shown), indicating a role of ATX in mitochondria ROS scavenging. Prior studies showed that the Nrf2 anti-oxidant response element (ARE) pathway plays an important role in the anti-oxidative effects of ATX [37]. Consistent with previous studies, our results showed that ATX upregulated NRF2 expression in irradiated c-kit-positive cells. Furthermore, the NRF2-targeted proteins HO-1 and NQO1, and anti-oxidative enzymes SOD2, CAT, and GPX1, were significantly upregulated in irradiated c-kit-positive cells in the presence of ATX (Fig. 11). To further explore the important role of Nrf2, *Nrf2* ^*−/−*^ mice were employed in the absence and presence of ATX. Results showed that ATX also decreased ROS level in c-kit-positive cells of *Nrf2* ^*−/−*^ mice after TBI at 4 Gy. These findings suggested that the radioprotective effects of ATX are partly dependent on the regulation of Nrf2 and its targeted proteins.

Previous studies have shown that overexpression of an anti-apoptotic protein or downregulation of a pro-apoptotic protein reduced IR-induced inhibition of hematopoietic function [5]. Whether ATX attenuates TBI-induced BM suppression by regulating the expression of these apoptotic-related proteins has yet to be investigated. Therefore, we used Western blot analysis to evaluate the expression of apoptotic-related proteins in c-kit-positive cells. Our results indicated that ATX-mediated inhibition of hematopoietic cell apoptosis is dependent upon upregulation of the BCL-XL protein and dow-regulation of BAX and BAK, members of the Bcl-2 family that are involved in the apoptotic signaling pathway. ATX reduced apoptosis in c-kit-positive cells, revealing a new anti-radiation property of this anti-oxidant. Song and colleagues suggested that ATX inhibits apoptosis in AECs-II via the ROS-dependent mitochondrial signaling pathway, which is involved in inhibition of cytochrome C and caspase-3 release, and activation of other cytoprotective genes [25]. Our similar results showed that ATX reduced cytochrome C release and the level of activated CASPASE 3 expression following irradiation. Therefore, ATX attenuates BM failure by inhibiting hematopoietic cell apoptosis via regulating the intrinsic apoptotic pathway (Fig. 11).

In addition, IR is extensively used to treat lung cancer and malignant pelvic cancers, but the toxicity to the surrounding healthy tissues limits the application of radiotherapy. Radiation-induced lung fibrosis is one of the most serious effects of lung cancer radiotherapy on normal tissue, and gastrointestinal radiation toxicity is found in the majority of patients treated for pelvic cancers [28, 38, 39]. Because ATX has been reported to ameliorate bleomycin-induced pulmonary fibrosis in vitro and in vivo [28], it will be interesting to determine whether ATX can also ameliorate pulmonary fibrosis caused by IR. Furthermore, the dose of radiation that leads to the gastrointestinal syndrome was higher than that required for inducing the hematopoietic syndrome [40, 41]; in the future, we will explore the effect of ATX on gastrointestinal radiation toxicity in abdominal irradiation models. ATX may thus have multiple therapeutic uses for patients undergoing tumor radiotherapy.

## Conclusions

Our results demonstrate that TBI induces apoptosis in c-kit-positive cells and generates ROS, which lead to hematopoietic cell depletion and destruction of the BM reconstitution potential. Owing to its superior anti-oxidant properties, ATX significantly ameliorates TBI-induced BM suppression by inhibiting apoptosis of hematopoietic cells and scavenging intracellular ROS. These findings suggest a new strategy for protecting the hematopoietic system against IR.

## Abbreviations

ATX: Astaxanthin
BM: Bone marrow
CAT: Catalase
CFU-GM: Colony of granulocyte macrophage cells
DCFDA: 2′, 7′-dichlorodihydrofluorescein diacetate
DHE: Dihydroethidium
DSB: double-strand break
GPX1: Glutathione peroxidase 1
HO-1: Heme oxygenase-1
HPC: Hematopoietic progenitor cell
HSC: Hematopoietic stem cell
IR: Ionizing radiation
Keap1: Kelch-like ECH-associated protein 1
LSK: Lin^−^Sca-l^+^c-kit^+^
MFI: Mean fluorescence intensity
NQO1: NAD(P)H quinone oxidoreductases 1
Nrf2: Nuclear factor erythroid 2-related factor
PBS: Phosphate-buffered saline
ROS: Reactive oxygen species
SOD: Superoxide dismutase
TBI: Total body irradiation
WBC: White blood cell
